# Schip1 Is a Novel Podocyte Foot Process Protein that Mediates Actin Cytoskeleton Rearrangements and Forms a Complex with Nherf2 and Ezrin

**DOI:** 10.1371/journal.pone.0122067

**Published:** 2015-03-25

**Authors:** Ljubica Perisic, Patricia Q. Rodriguez, Kjell Hultenby, Ying Sun, Mark Lal, Christer Betsholtz, Mathias Uhlén, Annika Wernerson, Ulf Hedin, Timo Pikkarainen, Karl Tryggvason, Jaakko Patrakka

**Affiliations:** 1 Division of Matrix Biology, Department of Medical Biochemistry and Biophysics, Karolinska Institute, Stockholm, Sweden; 2 Clinical Research Center, Department of Laboratory Medicine, Karolinska Institute, Stockholm, Sweden; 3 Vascular Biology Division, Department of Medical Biochemistry and Biophysics, Karolinska Institute, Stockholm, Sweden; 4 Department of Biotechnology, Royal Institute of Technology, Stockholm, Sweden; 5 Division of Renal Medicine, Department of Clinical Science, Intervention and Technology, Karolinska Institute, Stockholm, Sweden; 6 Division of Vascular Surgery, Department of Molecular Medicine and Surgery, Karolinska Institute, Stockholm, Sweden; Fondazione IRCCS Ospedale Maggiore Policlinico & Fondazione D’Amico per la Ricerca sulle Malattie Renali, ITALY

## Abstract

**Background:**

Podocyte foot process effacement accompanied by actin cytoskeleton rearrangements is a cardinal feature of many progressive human proteinuric diseases.

**Results:**

By microarray profiling of mouse glomerulus, SCHIP1 emerged as one of the most highly enriched transcripts. We detected Schip1 protein in the kidney glomerulus, specifically in podocytes foot processes. Functionally, Schip1 inactivation in zebrafish by morpholino knock-down results in foot process disorganization and podocyte loss leading to proteinuria. In cultured podocytes Schip1 localizes to cortical actin-rich regions of lamellipodia, where it forms a complex with Nherf2 and ezrin, proteins known to participate in actin remodeling stimulated by PDGFβ signaling. Mechanistically, overexpression of Schip1 in vitro causes accumulation of cortical F-actin with dissolution of transversal stress fibers and promotes cell migration in response to PDGF-BB stimulation. Upon actin disassembly by latrunculin A treatment, Schip1 remains associated with the residual F-actin-containing structures, suggesting a functional connection with actin cytoskeleton possibly via its interaction partners. A similar assay with cytochalasin D points to stabilization of cortical actin cytoskeleton in Schip1 overexpressing cells by attenuation of actin depolymerisation.

**Conclusions:**

Schip1 is a novel glomerular protein predominantly expressed in podocytes, necessary for the zebrafish pronephros development and function. Schip1 associates with the cortical actin cytoskeleton network and modulates its dynamics in response to PDGF signaling *via* interaction with the Nherf2/ezrin complex. Its implication in proteinuric diseases remains to be further investigated.

## Introduction

The kidney glomerulus is a tuft of specialized capillaries with a fairly high-pressure flow, endowing the ability to filter large amounts of water and small solutes into the urinary space, while retaining albumin and bigger molecular size proteins [1]. The glomerular filtration barrier (GFB) is composed of fenestrated endothelial cells that line the capillary loops, a glomerular basement membrane (GBM) and podocytes that cover the outside of the GBM. Over the past decades, the study of podocytes has revealed many novel facets of glomerular disease [2,3]. The cytoarchitecture of podocytes is defined by three principal compartments: the cell body, the major processes and the interdigitating foot processes. In order to withstand high pressure in the capillaries, the podocyte must possess a dynamic contractile apparatus, and to maintain intact and exact filtration properties, the arrangement of the cytoskeleton needs to be precisely spatially and temporally controlled [2]. Actin filaments are the predominant cytoskeletal components of podocyte foot processes [4] and this actin network contains a unique assembly of linker and adaptor molecules [5,6]. Besides acting as a scaffold for submembrane protein complexes, the cortical actin cytoskeleton provides a tensile architectural support for podocyte cellular extensions.

Nephropathies characterized by podocyte dysfunction, represent some of the most common causes of renal replacement [7]. The histopathological features include glomerulosclerosis, podocyte foot process effacement and GBM thickening. At the molecular level, it has been recognized that proteins participating in the regulation of podocyte actin organization may represent potential targets in the treatment of these diseases [8].

We have previously analyzed the mouse glomerular transcriptome in detail [9]. One of the highly glomerulus-enriched transcripts encoded for Schip1 (Schwannomin interacting protein 1), a relatively uncharacterized protein discovered originally through its interaction with the tumor suppressor Merlin (neurofibromatosis type 2, schwannomin) in the mouse brain [10]. Two splice isoforms of Schip1 exist and in the brain it is also found as a product of the fusion gene named IQCJ-SCHIP1. This is a complex transcriptional unit that bridges two separate genes IQCJ and Schip1, and it has been shown that the fusion protein localizes to mature nodes of Ranvier and axons [11]. Schip1 null-knockout mice generated by Schmahl et al. [12] exhibit skeletal, craniofacial and digestive problems, but no overt kidney phenotype. However, Schip1-/-PDGFrβ+/- mice show decreased kidney function manifested as swollen and degraded glomeruli, originally attributed to mesangial cell defects.

Schip1 interaction partner Merlin belongs to the family of ERM proteins (Ezrin, Radixin and Moesin) that function not only as linkers between the cell membrane and the cortical cytoskeleton, but also as active modulators of the actin cytoskeleton and membrane receptors in the subcortical areas of cell extensions [13]. In the podocyte, ezrin has been identified as a linker between the apical membrane glycoprotein podocalyxin and the actin cytoskeleton [6]. Ezrin binds to Nherf2 and the integrity of this complex seems to be important for the maintenance of podocyte foot processes and functional filtration barrier [6,14].

Here we characterized the role of Schip1 by analyzing its expression pattern in kidney tissue, functionally by inactivating its expression in zebrafish and mechanistically by investigating its protein interaction network and role in cultured cells. Our results highlight Schip1 as a novel glomerular protein predominantly expressed in podocytes, necessary for the zebrafish pronephros development. Schip1 associates with the cortical actin cytoskeleton network and modulates its dynamics in response to PDGF signaling *via* interaction with the Nherf2/ezrin complex.

## Results

### Schip1 is expressed in kidney glomerulus and localizes to podocyte foot processes

**Fig 1:**
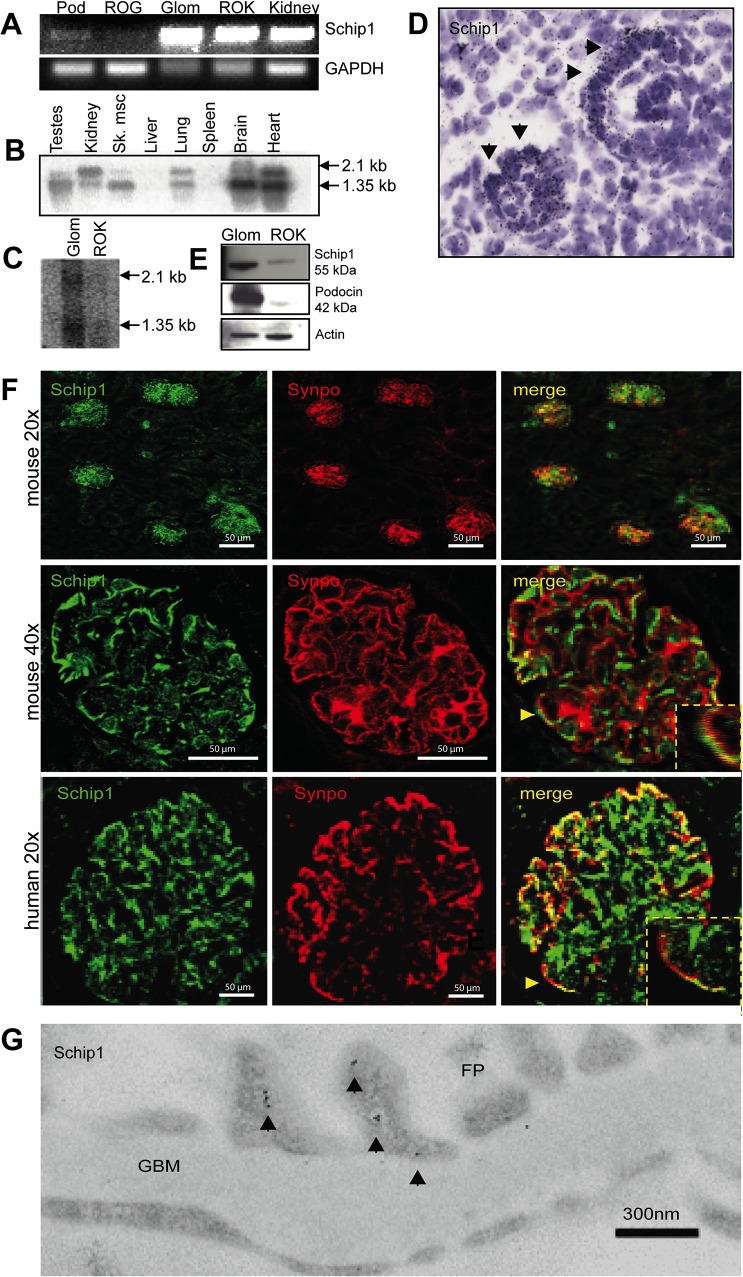
Schip1 is expressed by glomerular podocytes. **(A)** RT-PCR shows SCHIP1 expression in both glomerulus and the kidney fraction lacking glomeruli. In the glomerulus, expression is mostly detected in FACS-sorted podocytes. As an internal control, expression levels of GAPDH were measured. Controls for markers of various fractions are presented in S3 Fig. (Pod-podocytes, ROG-rest of glomerulus, GLOM-glomerulus, ROK-rest of kidney). **(B)** Northern blotting on a mouse multiple tissue panel shows the presence of two SCHIP1 mRNA transcripts enriched in the brain, heart, testes and kidney tissues. **(C)** Northern blotting on mouse glomerulus and ROK tissue confirms stronger SCHIP1 expression in the glomerulus, and presence of two transcripts. **(D)** By radioactive *in situ* hybridization on newborn mouse kidney sections SCHIP1 mRNA is localized to developing podocytes of the capillary loop stage glomerulus. **(E)** By Western blotting, the mouse 55kDa Schip1 protein is detected mostly in the glomerulus. Podocin was used as a positive control for the glomerular fraction, β-actin as a loading control. **(F)** Immunofluorescence on mouse and human kidney sections shows Schip1 glomerulus signal that partially overlaps with a podocyte foot process marker synaptopodin (Synpo). **(G)** By immunoelectron microscopy, Schip1 localizes to the glomerular podocyte foot processes (FP) in human kidney sections (GBM-glomerular basement membrane).

Previously, we observed that SCHIP1 transcript is enriched in the mouse kidney glomerulus as compared to rest-of-kidney (ROK) tissue (2.17 fold, p = 0.00443) by microarray profiling [9]. Here, we first confirmed the presence of SCHIP1 transcript in the kidney and the glomerulus fraction by RT-PCR and Northern blotting (Fig. 1A, B, C). SCHIP1 mRNA was mostly expressed by freshly isolated glomerular podocytes, while only weak signal was amplified from the rest-of-glomerulus (ROG). The mRNA expression pattern was confirmed by *in situ* hybridization in developing glomeruli (Fig. 1D). By Western blotting, anti-Schip1 antibody recognized an expected 55kDa protein in glomerular fractions whereas a weak band was detected in the kidney fraction devoid of glomeruli (Fig. 1E). To localize the Schip1 protein in the kidney, we double-stained adult mouse and human kidney tissues with anti-Schip1 and anti-synaptopodin antibodies. Schip1 localized to glomerulus, where the signal partially overlapped with that of the foot process marker synaptopodin (Fig. 1F). Limited overlap was seen with endothelial and mesangial cell markers (CD31 and aSMA, respectively, S3 Fig.). By immunoelectron microscopy (IEM) on normal human kidney sections, the majority of labeling for Schip1 (roughly 70%) was found in podocyte foot processes, where it localized mostly in the vicinity of the plasma membrane laterally, apically and basally (Fig. 1G and S3 Fig.). Labeling was detected also in endothelial cells (about 15%) and a minor amount in mesangial cells.

**Fig 2:**
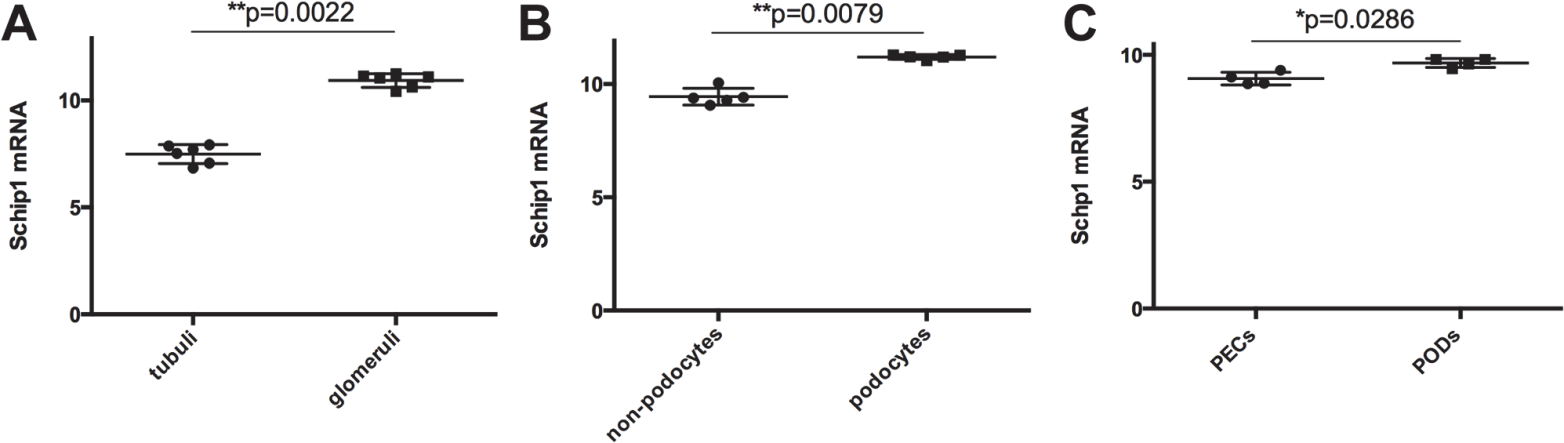
Additional evidence of Schip1 expression in podocytes. SCHIP1 is significantly upregulated in microarrays from human glomeruli vs. tubuli comparison **(A)**, in mouse podocytes vs. non-podocyte cells **(B)** and in mouse podocytes (visceral epithelial cells) vs. parietal epithelial cells **(C).**

Seeking to validate our results regarding SCHIP1 expression we searched the ArrayExpress (www.ebi.ac.uk/arrayexpress) repository for other public microarray datasets similarly focusing on the expression profile of the kidney glomerulus. In support of our results, SCHIP1 was found to be significantly upregulated in human glomeruli vs. tubuli fraction (mean difference between expression levels with standard deviation (SD) = 3.443±0.2238, p = 0.0022; dataset GSE21785, Fig. 2A)[15], in mouse podocytes vs. non-podocyte glomerular cells (mean difference with SD = 1.759±0.1711, p = 0.0079; GSE39441, Fig. 2B)[16] as well as in primary mouse podocytes vs. parietal epithelial cells (mean difference with SD = 0.6189±0.1533, p = 0.0286; GSE33714, Fig. 2C)[17].

### Inactivation of Schip1 in zebrafish results in distorted podocyte foot process structure and leakage of the filtration barrier

**Fig 3:**
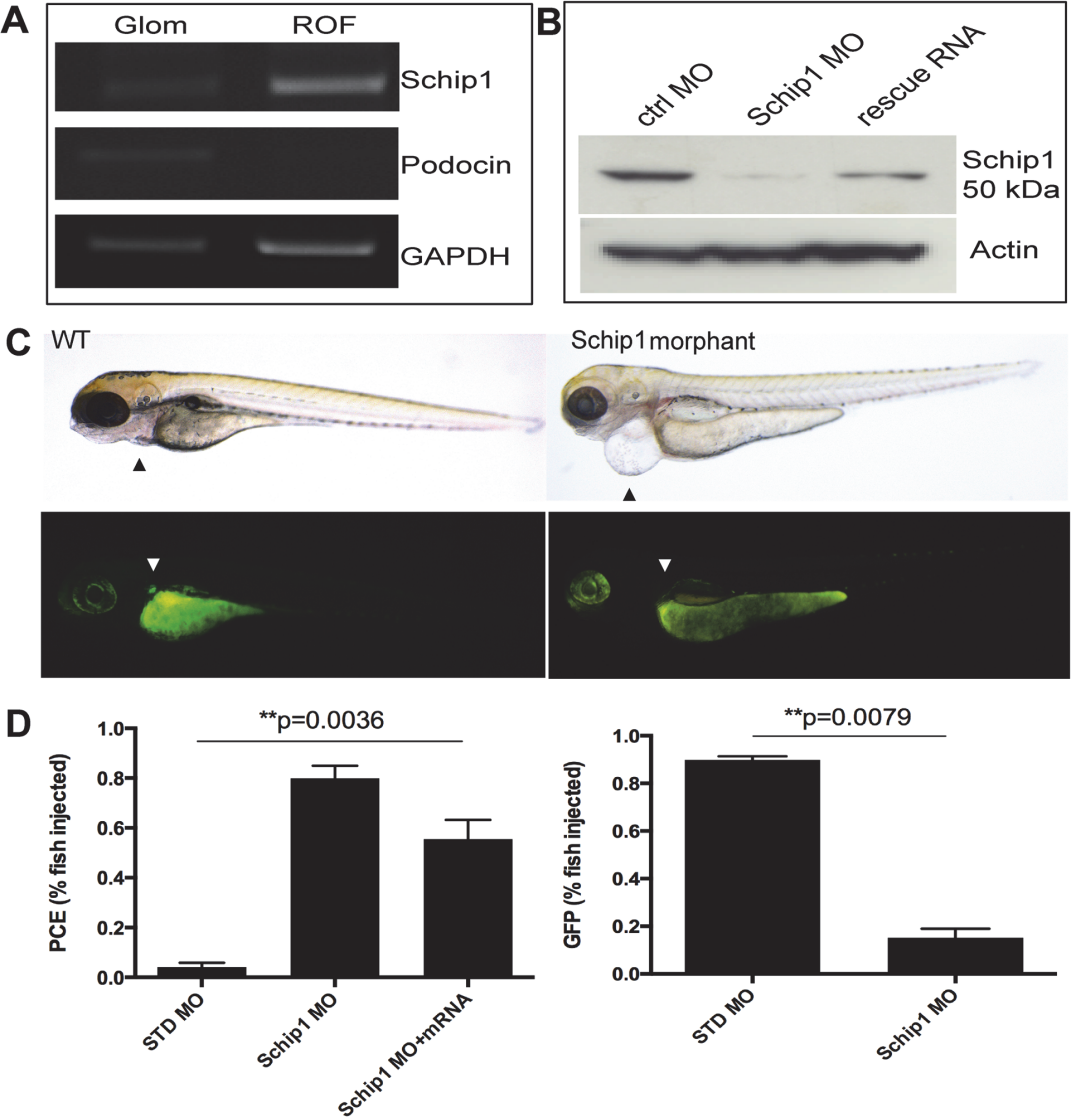
Schip1 is expressed in zebrafish pronephros and its inactivation leads to pericardial edema and loss of podocyte specific GFP-expression. **(A)** To confirm the presence of Schip1 in zebrafish, pronephroi were microdissected from the zebrafish line expressing the GFP under podocin promoter. RT-PCR from pronephros (Glom) and rest-of-fish (ROF) fractions shows the signal for Schip1 in pronephros. Podocin was used as the positive control to validate the purity of the pronephros fraction and GAPDH as the loading control. **(B)** Morpholino injection in zebrafish resulted in the reduction of Schip1 protein as confirmed by Western blot. An increase in Schip1 protein is detected in fish rescued by coinjection with mouse full-length Schip1 mRNA. Zebrafish Schip1 protein band 50kDa. Actin-beta used as loading control. **(C)** Inactivation of Schip1 led to development of pericardial edema in 96 hpf morphant embryos (upper panels, arrowheads). In podocin-GFP zebrafish line, Schip1 injection caused loss of GFP signal in pronephros (lower panels, arrowheads). **(D)** Graphs showing quantification of the zebrafish phenotypes. Data presented as mean with SEM of several experiments. WT-wild type, MO-morpholino, PCE-pericardial edema, ctrl-control, GFP-green fluorescent protein.

We used zebrafish as a model to study the function of Schip1 *in vivo*. Based on Ensembl database, zebrafish genome contains only one Schip1 orthologue (ENSDARG00000035175). Sequence identity between the zebrafish, mouse and human Schip1 at the amino acid level was about 90%, while the C-terminal part of Schip1 encoding the functional coiled-coil domains, was nearly identical (S1 Fig.). To confirm the presence of Schip1 orthologue in the zebrafish pronephros, we microdissected pronephroi from the zebrafish line expressing GFP in podocytes [18]. RT-PCR (Fig. 3A, left panel) analyses showed the presence of Schip1 transcript in the pronephros fraction. The purity of this fraction was validated using highly podocyte specific gene podocin as control. Podocin transcript was detected solely in the pronephros (Fig. 3A), whereas GAPDH was strongly expressed in both zebrafish isolates.

Schip1 expression was inactivated in zebrafish using antisense morpholinos designed against the transcription initiation site for Schip1 coding sequence. In total, n = 164 embryos were injected with control and n = 221 with Schip1 morpholinos, experiments were repeated 5 times. Morpholinos were injected at 150mM concentration and larvae analyzed at 72 and 96 hours-post-fertilization (hpf). Morpholino injection in wild-type TL zebrafish line resulted in the decrease of Schip1 protein as detected by Western blotting in whole fish lysates (Fig. 3B). Of embryos injected with Schip1 morpholino (morphants), 80±9% developed pericardial edema (PCE), whereas of embryos injected with control morpholino only 4±3% exhibited edema (Fig. 3C upper panels, Fig. 3D left graph). Of note, overexpression of Schip1 by mRNA injection in wild-type zebrafish did not produce any obvious effect. To establish the specificity of morpholino effects, we analyzed whether Schip1 morphant phenotypes could be rescued by co-injection of a full-length mouse Schip1 mRNA. In total n = 275 embryos were injected with Schip1 mRNA (75pg/nl) while n = 158 embryos were co-injected with Schip1 morpholino and mRNA (experiments repeated 5 times; results confirmed by Western blot, Fig. 3B). Indeed, in this fashion we were able to fully rescue the cardiac edema phenotype in 25±13% of embryos (Fig. 3D left), while a higher proportion (additional 50%) showed different levels of PCE reduction. The result indicates that the observed phenotype is specific to the knockdown of Schip1 expression.

Cardiac edema may result from renal dysfunction but may also reflect direct heart problems. To confirm the pronephros injury, we injected morpholinos to a zebrafish line expressing GFP specifically in podocytes [18]. The signal for GFP was detected only in 15±8% of Schip1 morphants, whereas 90±3% of control fish exhibited detectable GFP signal (Fig. 3C lower panels, Fig. 3D right graph).

**Fig 4:**
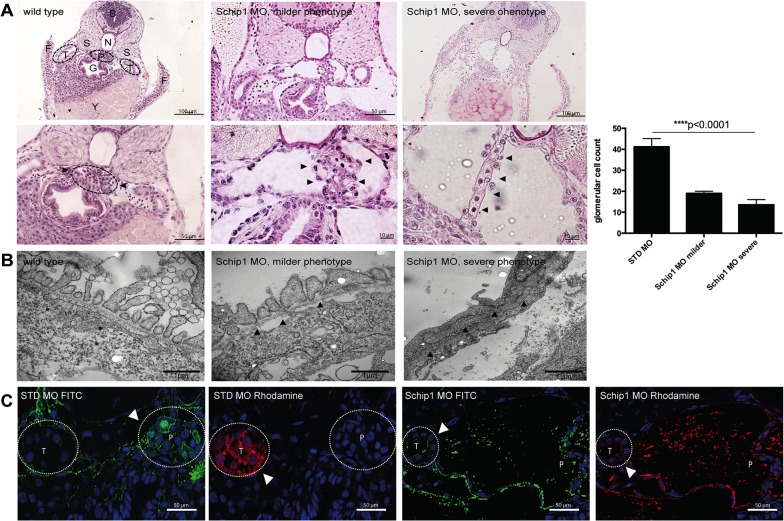
Schip1 inactivation in zebrafish causes distortion of podocyte foot processes, podocytopenia and leakage of the filtration barrier. **(A)** Brightfield images of PAS stained histological sections showing dilated Bowman’s space and distortion of proper podocyte structures (arrowheads) in Schip1 morphant zebrafish with milder (second panel) and more severe phenotype (third panel). Higher magnifications (size bars) shown in lower panel, including a cell nuclei quantification graph. **(B)** Electron microscopic analyses of zebrafish morphants show effaced podocyte foot processes with various degrees of damage (middle, arrowheads and right). However, slit diaphragms are consistently present (right, arrowheads). **(C)** Dye filtration assay in control and Schip1 zebrafish morphants. Rhodamine conjugated 10kDa is freely filtered into the tubuli of both control and morphant fish, whereas FITC labeled 500kDa dye remains in the pronephros in control embryos. In Schip1 morphants 500kDa dye accumulates in the Bowman’s space and also leaks into the tubuli. STD-standard control morpholino, F-fins, T-nephric tubuli, S-somites, B-brain, N-notochord, P-pronephros, G-gut, Y-yolk.

Histological sections of Schip1 morphant embryos showed that the pronephros exhibited dilated Bowman’s space and fewer podocytes (Fig. 4A, middle panels). In more severe cases, Bowman’s space was extremely dilated and in advanced stage glomerulus tuft was depleted. By transmission electron microscopy, the Schip1 morphants displayed a reduced number of cells in pronephros as quantified by counting nuclei (Fig. 4A, graph); those remaining podocytes exhibited effaced foot processes with intact slit diaphragms (Fig. 4B, arrowheads) while GBM and endothelial cells appeared normal. To assess the functionality of the Schip1 knockdown in zebrafish pronephros we performed the filtration assay mimicking human proteinuria by injecting 10kDa Rhodamine and 500kDa FITC Dextran dyes into the common cardinal vein. For this experiment n = 25 control and morphant larvae were injected, experiments were repeated 5 times and effects inspected at 80hpf. In control embryos injected with the standard control (STD) morpholino 500kDa dye was retained in the pronephros and no signal for FITC was seen in the tubuli (Fig. 4C, first panel), while 10kDa dye was filtered out into the tubuli (Fig. 4C, second panel). None of the control embryos displayed any filtration defect. Of Schip1 morphants 85,7% demonstrated filtration defects where both 10kDa and 500 kDa dyes could be detected in the tubular epithelial cells (Fig. 4C, third and fourth panels). However, both dyes accumulated abundantly also in the pronephros and the dilated Bowman’s space, suggestive of the defective filtration barrier. Taken together, the inactivation of Schip1 in zebrafish resulted in podocyte foot process effacement and podocytopenia leading to severe proteinuria.

### Schip1 associates with the cortical actin cytoskeleton

**Fig 5:**
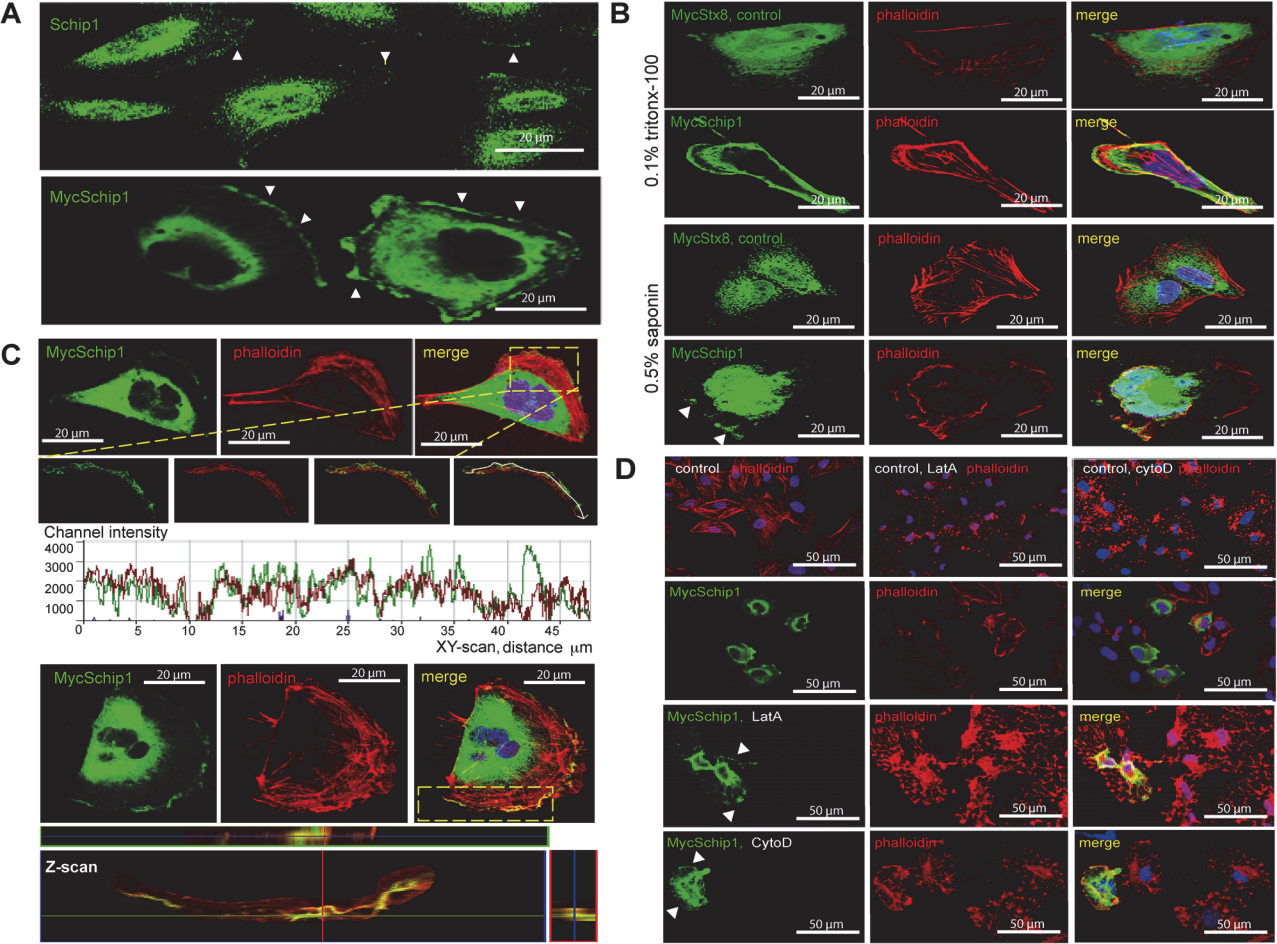
Schip1 localizes to cell lamellipodia and associates with the cortical actin cytoskeleton. **(A)** Both endogenous (arrowheads, upper panel) and ectopic (arrowheads, lower panel) Schip1 localize to lamellipodia in cultured human podocytes. **(B)** To test Schip1 association to actin-rich lamellipodia regions, transiently transfected human podocytes were treated with the standard procedure (fixation and Triton X-100 permeabilization, upper panel), or incubated with saponin prior to fixation and staining for MycSchip1 (lower panel). Peripheral Schip1 expression is partially retained after saponin treatment, indicating association of the protein with detergent-insoluble cytoskeletal/plasma membrane structures. The same was not observed in control cells transfected with Stx8 (syntaxin 8). **(C)** Schip1 colocalizes with cortical F-actin in the podocyte lamellipodia. Both the Z- and XY-scanning indicate considerable signal overlap between Schip1 and F-actin along the plasma membrane in cells presenting well-developed lamellipodia. **(D)** Treatment with latrunculin A results in the dissolution of actin fibers in both control and Schip1-transfected podocytes. However, Schip1 signal remains associated with disturbed F-actin positive residues. In contrast, treatment with cytochalasin D results mostly in preservation of the cortical actin in Schip1 transfected podocytes.

We then looked at cultured podocytes to get further insights into the mechanism of Schip1 function. Both endogenously (Fig. 5A upper panel and S2 Fig.) and ectopically expressed Myc-tagged Schip1 (Fig. 5A lower panel and S2 Fig.) were observed at lamellipodial extensions towards the cellular periphery. To confirm the lamellipodial localization we permeabilized Schip1-expressing cells with 0.5% saponin prior to fixation and immunostaining. This permeabilization strategy results in the removal of the soluble pool of proteins while sparing those that are more stably anchored. Under these conditions we found that Schip1 staining was partially retained, supporting the association with detergent-insoluble cytoskeletal/membrane structures (Fig. 5B, arrowheads). Examination of peripheral Schip1 by confocal microscopy further indicated a significant overlap with the cortical actin cytoskeleton both in the XY- and Z-scan (Fig. 5C). To determine the possibility of Schip1 association with the actin cytoskeleton at the cell cortex/lamellipodia, we treated the cells with latrunculin A, an actin monomer-sequestering drug that distorts the organization of cytoskeletal structures (Fig. 5D, S2 Fig.). Latrunculin A-treatment resulted in a loss of parallel transversal stress fibers as well as cortical actin filaments in both control and Schip1-transfected cells, with appearance of phalloidin-positive patches containing remnant actin fibers. Schip1 signal in transfected cells overlapped with this phalloidin staining (arrowheads), supporting the notion that the protein associates with residual actin fibers. To further examine the possibility of Schip1 being involved in the actin assembly mechanisms, we performed a similar assay with cytochalasin D, a potent inhibitor of actin polymerization. In this experiment, Schip1 overexpressing cells mostly maintained the proper actin filament structure especially at the cell-periphery where Schip1 was localized (Fig. 5D and S2 Fig.). Notably, nocodazole, a microtubule cytoskeleton disassembly drug, had no effect whatsoever on Schip1 cellular localisation (S2 Fig.). Thus, it appeared that Schip1 promotes stabilization of cortical F-actin by interfering with actin polymerization. The protein may be directly or indirectly associated with actin, nevertheless proper actin cytoskeleton structure is critically important for the localization of Schip1.

### Schip1 controls actin dynamics and chemokinesis in response to PDGF signaling

**Fig 6:**
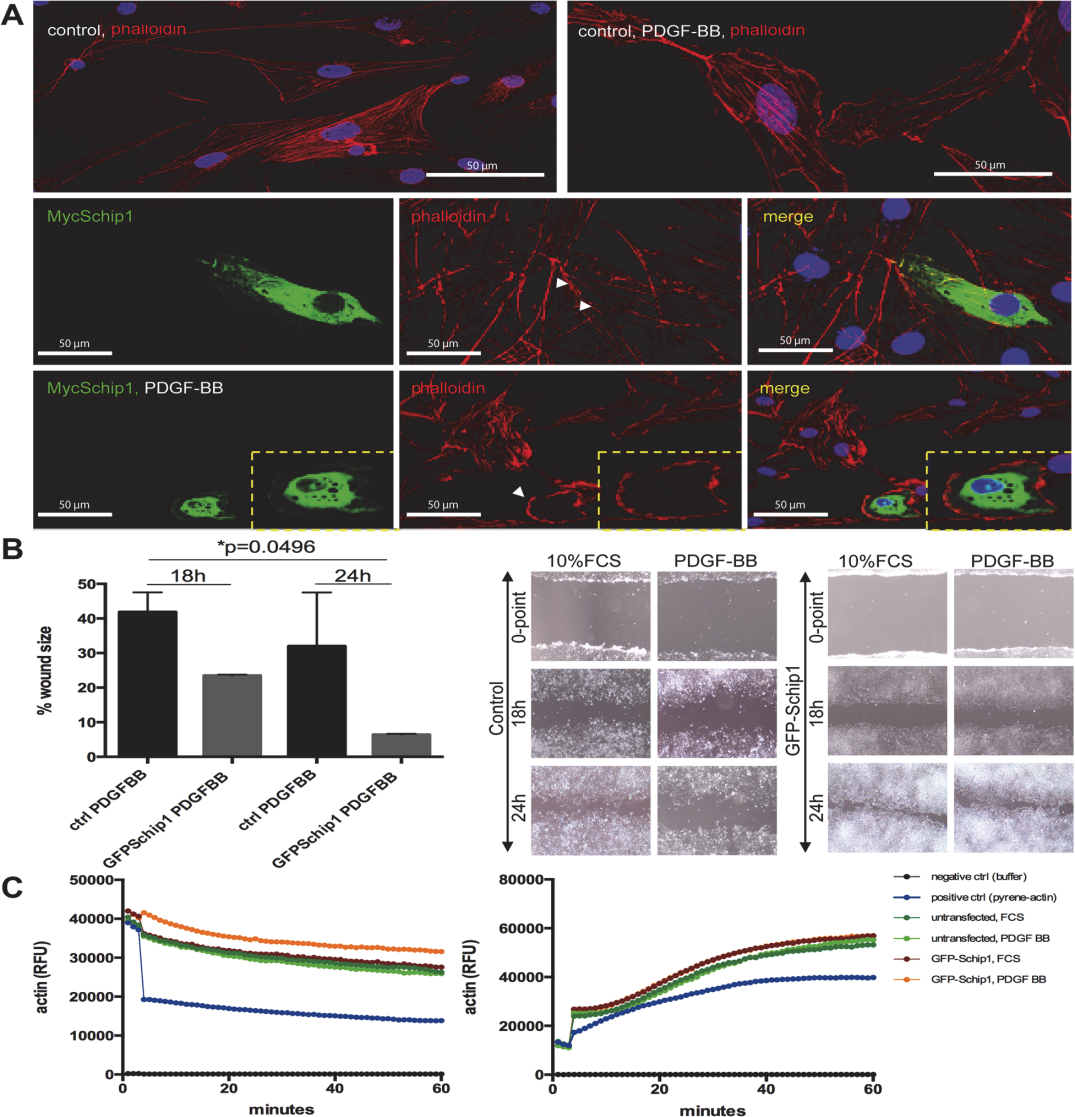
Schip1 overexpression promotes cortical F-actin accumulation, dissolution of stress fibers and motility in PDGF-BB-stimulated cells by attenuating actin depolymerisation. **(A)** In control cells PDGF-BB treatment enhances development of lamellipodia. In Schip1-transfected cells PDGF-BB stimulation induces similar changes but also marked actin cytoskeleton rearrangement with cortical actin accumulation and dissolution of the actin stress fibers (box, zoom). Observe the neighboring non-Myc-Schip1 expressing cells, presenting with normal actin cytoskeleton and pronounced stress fibers. **(B)** Stable Schip1-expressing and control HEK293 cells were stimulated with 10% FCS or PDGF-BB, scratched, and left to migrate for 24 h (the wound healing assay). Schip1 transfected cells exhibit similar migration rate as controls in medium supplemented with 10% FCS, but migrate faster when induced with PDGF-BB (graph). Microscopic images of control and Schip1-expressing cell monolayers 18 and 24 h after wound scratching (left). **(C)** In vitro actin polymerization (right panel) and depolymerization (left panel) assays with lysates from GFP–Schip1-expressing HEK293 cells and controls show that Schip1-overexpression slows down actin depolymerization in presence of PDGFBB in comparison to cells treated with 10% FCS (p<0.0001). Results are representative of three separate experiments. RFU-relative fluorescence units.

Since Schip1 has been highlighted as an early response gene in the PDGFβ signaling cascade [19], it was of interest to test the effect of PDGF stimulation on Schip1-overexpressing podocytes (Fig. 6). In control mock transfected cells PDGF-BB stimulation caused formation of large lamellipodial structures, with retention of parallel actin stress fibers throughout the cell body (Fig. 6A). In contrast, a 2-h stimulation of Schip1-transfected cells with PDGF-BB often led to accumulation of cortical actin with a loss of parallel actin stress fibers (Fig. 6A and S3 Fig.). In order to investigate whether the PDGF stimulation has Schip1-dependent functional consequences, we performed migration assays with stable GFP-Schip1-expressing HEK293 cells and control cells grown on fibronectin (Fig. 6B). The two cell populations exhibited similar migration rates in media containing 10% FCS. In contrast, when stimulated with PDGF-BB Schip1-expressing cells migrated faster than the controls (p = 0.0496). Combined with the previous finding that Schip1 stabilized actin cytoskeleton in cells treated with cytochalasin D, we decided to examine the behavior of Schip1-overexpressing cells in actin polymerization and severing assays (Fig. 6C). Interestingly, actin depolymerisation (severing) was significantly slower (p<0.0001) in Schip1-overexpressing cells stimulated with PDGF-BB compared to those treated with 10% FCS, whereas no difference in actin polymerization was detected. Hence, it appears that Schip1 promotes cell migration in response to PDGF signaling through its effects on the actin cytoskeleton rearrangements by attenuating actin depolymerisation.

### Schip1 interacts with Nherf2 and ezrin, a complex that links actin cytoskeleton to the plasma membrane

**Fig 7:**
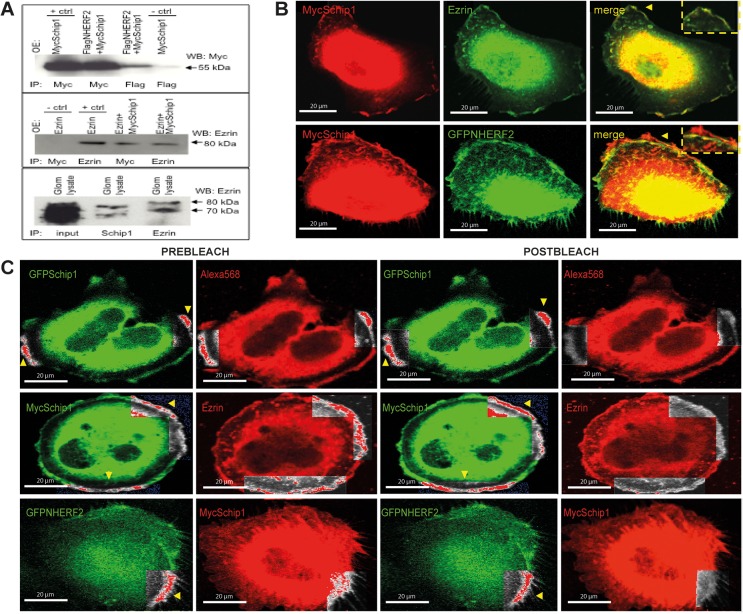
Schip1 interacts and colocalizes with Nherf2 and ezrin *in vitro*. **(A)** Myc-tagged Schip1 and Flag-tagged Nherf2 coimmunoprecipitate from lysates of cotransfected HEK293 cells (upper panel). Negative control (MycSchip1 transfection, Flag IP, anti-Myc blot) is shown in lane 4 and positive control (MycSchip transfection, Myc IP, anti-Myc blot) in lane 1. MycSchip1 also interacts with ezrin, a protein known to be in complex with Nherf2, in cotransfected HEK293 (middle panel). Negative control (Ezrin transfection, Myc IP, anti-Ezrin blot) is here in lane 1 and positive control (Ezrin transfection, Ezrin IP, anti-Ezrin blot) in lane 2. Endogenous Schip1 and ezrin interact in pig glomerular lysates as shown by coimmunoprecipitation (lower panel). Whole blots are presented in S4 Fig. OE-overexpression by transfection, IP-immunoprecipitation, WB-Western blot. **(B)** Schip1 colocalizes with ezrin and Nherf2 in cultured human podocytes to cortical actin zones and lamellipodia. Schip1 and ezrin show very close overlap (arrowheads, boxed area, zoom), whereas Schip1 and Nherf2 colocalize partially (boxed area, zoom). **(C)** Interaction of Schip1 with Nherf2 and ezrin was confirmed by FRET in cotransfected, fixed podocytes. MycSchip1 signal intensity increases upon Alexa568 bleaching (boxed area, arrowheads) as a result of FRET between the two fluorophores (Alexa568 and Alexa488) suggesting associations of Schip1 with ezrin and Nherf2 proteins (middle and bottom panels). As a positive control, we used GFP-Schip1 stained with anti-GFP- and Alexa Fluor 568-conjugated secondary antibodies (upper panel). We detected about 40% FRET signal increase between Schip1 and ezrin. Lower FRET signal increase of about 10% was detected between Schip1 and Nherf2 (n = 20 ROIs tested in each experiment).

To identify the Schip1 protein interaction network and further elucidate the mechanism of its function, we performed yeast two-hybrid screens with Schip1 constructs as baits using a mouse glomerular cDNA library of preys. Using the Schip1 most C-terminal coiled-coil domain (amino-acids 398–459) as the bait, we identified the full-length Nherf2 as the potential interacting partner of Schip1. Interestingly, Nherf2 transcript is also enriched in the mouse glomerulus as compared to rest-of-kidney by microarray profiling (3.2 fold, p = 0.00336) [9]. Coimmunoprecipitation from transfected HEK293 cells confirmed the interaction between Schip1 and Nherf2 (Fig. 7A, upper panel, whole blot in S4 Fig.). As Nherf2 is known to form a complex with ezrin in podocytes, we tested whether Schip1 interacts also with this linker between the cortical actin cytoskeleton and the plasma membrane. As suspected, Schip1 and ezrin coimmunoprecipitated from transfected cells (Fig. 7A, middle panel, whole blot in S4 Fig.). Importantly, we were able to coprecipitate endogenous Schip1-ezrin interaction also from pig glomerular lysates (Fig. 7A, bottom panel, whole blot in S4 Fig.). In further support of these results, Schip1, ezrin and Nherf2 colocalised at the lamellipodia periphery in cultured podocytes (Fig. 7B). In addition to coprecipitations, we used Förster resonance energy transfer (FRET) coupled to confocal microscopy to validate the interactions of Schip1 with Nherf2 and ezrin in cultured human podocytes (Fig. 7C). In this experiment, we co-expressed Schip1, ezrin and Nherf2 proteins tagged at their N-termini with either GFP or Myc-tags, and measured the change in green donor fluorescence following bleaching of the red acceptor fluorophore. FRET signal was detected between Schip1 and ezrin in the cellular lamellipodia and between Schip1 and Nherf2, confirming the direct interaction of the proteins.

**Fig 8:**
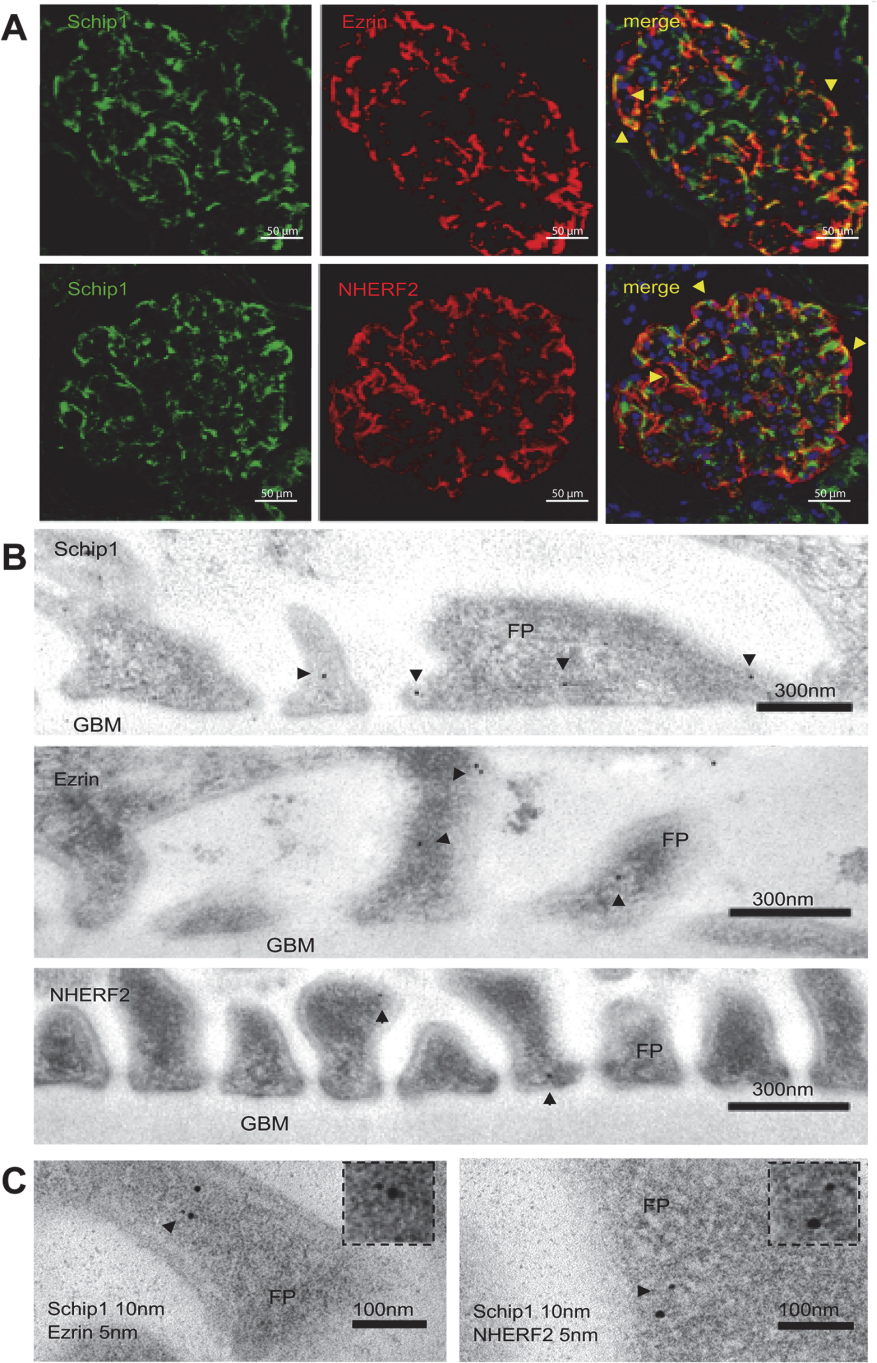
Schip1, ezrin and Nherf2 colocalize in the podocyte foot processes of the human kidney glomerulus. **(A)** Immunofluorescence on human kidney sections shows partial colocalization of Schip/ezrin and Schip/Nherf2 in the glomerulus (arrowheads). **(B)** By IEM, Schip1 is localized to the podocyte foot processes (FP), often to the apical but also to the basolateral side. Similar localization is seen for ezrin and Nherf2 (arrowheads). **(C)** Double IEM for Schip1 (10 nm gold particle) and ezrin or Nherf2 (5 nm gold particle) indicates that the proteins colocalize at the same subcellular area in the foot processes (arrowheads, zoom).

Next, we analyzed whether these proteins colocalize in the human glomerulus *in situ*. By immunofluorescence we observed a partial overlap of Schip1 with ezrin and Nherf2 signals in the glomerulus (Fig. 8A). IEM studies of Schip1 and ezrin/Nherf2 (labeled with 10 nm and 5 nm gold particles, respectively) indicated close colocalization of Schip1 and ezrin in podocytes foot processes within 15.65 ± 1.514 nm of each other (Fig. 8B and 8C, right panel, zoom). Schip1 and Nherf2 colocalized 21.25 ± 8.260 nm from each other (Fig. 8B and 8C, left panel, zoom). Altogether, our results provide solid evidence for the interaction of Schip1 with Nherf2 and ezrin.

## Discussion

We identified Schip1 as a novel, important molecular component of the podocyte foot processes. *In vivo*, inactivation of Schip1 in zebrafish leads to glomerular damage and proteinuria through podocyte foot process effacement. *In vitro*, we show that Schip1 interacts with Nherf2 and ezrin that directly bind to the cytoplasmic tail of podocalyxin [6,14], linking this apical membrane protein to the actin cytoskeleton. Recently, a protein named CLIC5A was also shown be a component of the podocalyxin-Nherf2-ezrin complex [20]. Schip1 could be another constituent of the same sub-membrane network *via* Nherf2-Ezrin, participating in the dynamics of actin cytoskeleton rearrangements. Previously, podocyte damage observed in protamine-sulfate, sialidaze or puromycin-aminonucleoside treated rats and in CLIC5A knockout mice, was shown to be caused by the disruption of this complex [14,20,21]. Most recently, Wasik et al. reported reduced protein levels of Nherf2 and ezrin in podocytes in both experimental nephrosis in rats and in patients with diabetic nephropathy [22]. Collectively, our results additionally underline the functional association among these proteins in the glomerular pathology.

We used zebrafish as a model to study the role of Schip1 in the glomerulus. Severe pronephros injury was observed in Schip1 zebrafish morphants, characterized by podocyte foot process effacement and podocyte loss leading to leakage in the filtration barrier as confirmed by the labeled dextran dye assay, indicating the importance of Schip1 for podocytes. The use of zebrafish in studies of gene function and kidney disease is nowadays well established [18,23–25], nevertheless it may present with certain challenges. Genes may have multiple copies in the zebrafish genome, it might be difficult to validate the orthologue expression in zebrafish, knockdowns could be problematic to verify, and morpholinos can have off-target effects. In case of SCHIP1, we were able to identify only a single zebrafish orthologue. We demonstrated its expression in zebrafish using RT-PCR and Western blotting, and the glomerulus expression was confirmed by isolating GFP-positive pronephroi. Because of the high level of amino-acid identity, our antibodies raised against the C-terminal part of the human Schip1 cross-reacted with the zebrafish protein, which enabled us to validate the inactivation of Schip1 at the protein level. Finally, to exclude off-target effects, we confirmed the specificity of our morpholino by rescuing the phenotype with mouse SCHIP1 mRNA. This experiment indicated that the phenotype was likely secondary to the loss of Schip1.

Foot process effacement and podocytopenia are cardinal features of many progressive human proteinuric diseases, such as diabetic nephropathy and glomerulosclerosis. It is noteworthy that Schip1 morphants have normal appearance and do not show signs of injury until after the pronephros has matured around 72–80hpf, which is when the pericardial edema becomes evident. Schip1 morphants at 120hpf have extensive pericardial edema accompanied by tubular edema and die shortly after. The pericardial edema appearing later in development is a typical symptom of damage in the filtration barrier. Based on the late pericardial edema, the GFP-podocin-negativity of the pronephros and the filtration barrier leakage together, we can infer that podocytes in Schip1 morphants are not intact and that edema develops secondary to the podocyte injury. It is also conceivable that the remaining podocytes in fish with milder phenotype are arrested in some earlier developmental stage and so the glomerular injury could be caused not only by podocytopenia but also by changes in embryonic development. The podocyte foot process effacement seen at the electron microscopic level is associated with actin cytoskeleton rearrangements and proteinuria in zebrafish as well as in human disease and murine models, as shown previously by many researchers [23,25]. Taken together, our studies in zebrafish indicate that Schip1 contributes to the normal podocyte function.

In cultured podocytes, we demonstrated that Schip1 concentrates to cortical actin-rich zones of lamellipodia, where it also associates with the actin cytoskeleton. The formation of cortical actin-enriched lamellipodia, membrane ruffles and filopodia in cultured cells, downstream of Rho/Rac GTPase activation, are cellular features characteristic of PDGF receptor stimulation [26]. We observed that Schip1 overexpression promoted F-actin rearrangement in PDGF-BB-treated cells, as well as stimulated cell migration in response to the growth factor. Similar results have previously been reported by Schmahl et al. [12], as they showed that embryonic fibroblasts isolated from *Schip1-/*- mice exhibit significantly decreased migration in response to PDGFrβ activation. Although *Schip1-/*- mice do not present overt kidney phenotype in general, genetic challenge by loss of one PDGFrβ allele in *Schip1-/- PDGFrβ+/*- mice leads to decreased kidney function [12]. In combination with other published data, it was suggested that Schip1 is one of the early response genes controlling specific processes downstream of PDGFβ signaling [19]. We propose that the apparent discrepancy in phenotype between the mouse *Schip1* knock-out and zebrafish knock-down comes from the fact that zebrafish as a lower organism has less compensation mechanisms and tends to display a phenotype more prominently than mouse models. Collectively, results from Schmahl et al. and us indicate that Schip1 acts in regulation of actin remodeling upon PDGFβ stimulation. Here, we show evidence that PDGFβ signaling can be mechanistically linked to Schip1 *via* its protein interaction network comprising Nherf2 and ezrin, discovered through an unbiased and glomerulus-focused approach. Nherf2 has been shown to interact directly with the intracellular tail of PDGFrβ [27], to potentiate the receptor activity upon stimulation with PDGF [28] and to play a role in subsequent actin reorganization [27]. Theisen et al. [29] have shown that Nherf2 also links N-cadherin/catenin complex localized in adherens-junctions to PDGFr and actin cytoskeleton, which is important in regulation of cell motility. This is interesting since it has been demonstrated that the expression of PDGFβ receptors in podocytes is upregulated during the progress of diabetic nephropathy [30]. Interaction with Nherf2 and ezrin and participation in the PDGFβ signaling axis highlights the possibility of Schip1 involvement in diabetic nephropathy, which should be the course for future functional studies of this protein.

## Conclusions

In summary, we have shown that Schip1 is a novel component of the kidney glomerular filter localized in podocytes foot processes, indispensable for the zebrafish pronephros development and function as shown by morpholino knock-down. We propose that Schip1 is an adaptor protein in complex with ezrin-Nherf2 that links actin cytoskeleton to the podocyte foot process plasma membrane and mediates its dynamics in response to PDGFβ. Further studies are needed to dissect the Schip1 implication in glomerular pathology.

## Material and Methods

### Human and animal material

For immunoelectron microscopic studies, normal renal tissue was taken from unaffected kidneys surgically removed because of the localized carcinoma. The number of subjects is limited to three. The study was approved by the Regional Ethical committee of Stockholm (number 2005/1395–32). For immunofluorescence stainings, human samples were taken from cadaver kidneys unsuitable for transplantation because of vascular abnormalities (from the 4^th^ Department of Surgery, Helsinki, Finland). The study was approved by the Ethical committee for children’s diseases and psychiatry in Helsinki (permit number 22/E7/2007). All human material was collected with written informed consent from patients, organ donors or their guardians and used in agreement with the local Board of Ethics. Animal studies were carried out at Scheelelaboratoriet (Karolinska Insitutet) and approved by the Committee on Research Animal Care of North Stockholm (Stockholms Norra djurförsöksetiska nämnd, permit number N250/11).

### Expression constructs

Inserts were amplified from kidney cDNA (Mouse MTC™ Panel I, Clontech, Palo Alto, CA). For yeast two-hybrid screening, inserts were cloned into the vector pGBKT7 (Clontech) in frame with the Gal4 DNA-binding domain. For expression in mammalian cells, cDNAs were cloned into pcDNA3.1 (Invitrogen, Carlsbad, CA), or vectors with various N-terminal tags (pCMV-Myc, pCMV-HA, or pEGFP-C; all from Clontech).

### Antibodies

Following commercial antibodies were used: rabbit polyclonal anti-Schip1 (Sigma Prestige Antibodies, St. Luis, MO), mouse polyclonal anti-Schip1 (Abcam Cambridge, UK), rabbit anti-SLC9A3R2 (Nherf2) (Sigma Prestige Antibodies), rabbit polyclonal anti-ezrin (Abcam), mouse monoclonal anti-ezrin (Zymed, San Francisco, CA), monoclonal mouse anti Myc-tag (Sigma), rabbit anti-Myc-tag (Sigma), rabbit polyclonal anti-HA (Sigma), mouse anti-synaptopodin (Progen, Germany), anti-GFP (Invitrogen), anti-calnexin (Abcam), anti-β-actin (Abcam), anti-podocin (Sigma). Proteins were visualized with secondary antibodies conjugated to various Alexa Fluor dyes (488, 546, 568; all from Invitrogen) or HRP (GE Healthcare, Piscataway, NJ, US). Phalloidin and DAPI were purchased from Molecular Probes.

### Reverse transcription (RT) and RT-PCR

To generate glomerular and “rest-of-kidney” cDNA, mouse glomeruli and kidney tissue devoid of glomeruli (rest-of-kidney) were isolated as previously described [9]. Fluorescently labeled podocytes were derived by FACS sorting of cells from glomeruli obtained from bitransgenic R26-stop-EYFP mice harboring the Cre transgene driven by the podocin promoter (purchased from Jackson Laboratories). Total RNA was then isolated by RNeasy mini Kit (Qiagen, Valencia, CA) and 1 μg was reverse transcribed by Superscript III Reverse Transcriptase (Invitrogen).

### Northern blot and *in situ* hybridization

For both Northern blot and in situ hybridization a specific 500 bp probe corresponding to the 3’UTR of Schip1 was amplified from glomerular cDNA library and prepared as previously described [31]. For Northern blot the probe was ^32^P-dCTP labeled with Prime-It RmT Random Primer Labeling Kit (Stratagene, La Jolla, CA) and hybridized with Mouse MTN Blot (Clontech). The blots were exposed to a PhosphorImager SF screen and analyzed with ImageQuant software (Molecular Dynamics). For *in situ* hybridization the same probe was cloned into pCR II-TOPO Dual Promoter Vector (Invitrogen). Hybridisations were performed on paraffin embedded mouse kidney tissue sections (10 μm) [31].

### Zebrafish knockdowns, histological analyses and dye filtration assay

Zebrafish were housed as previously reported [23] following European and Swedish animal husbandry guidelines. Morpholino injection using standard procedures were performed on wild type TL and Podocin-GFP transgenic fish embryos [18]. Zebrafish were injected with Standard Negative Control (5’- cctcttacctcagttacaatttata-3’) and Schip1 (5’- tgcccagatccatgccatcctcccg -3’) morpholinos purchased from Gene-Tools (Oregon, USA) as well as with mouse full length Schip1 mRNA for rescue experiments. Embryos were kept in E3 medium at 28.5°C and 24 hours post-fertilization (hpf) the medium was changed to E3 containing 1-phenyl-2-thiourea (PTU, Sigma Aldrich) at a concentration 0.003% w/v to inhibit pigment formation. Histological analyses were performed on JB-4 embedded larvae using PAS staining and imaging with light microscopy. Samples were collected at 72hpf and 96hpf and processed according to standard procedures. For dye filtration assay mimicking human proteinuria [23,32,33], 10kda Rhodamine Dextran Dye and 500kDa FITC Dextran dissolved in 0.2KCl (1% total concentration each) were injected into the common cardinal vein. Assay was performed in 80hpf embryos for 2hrs after which they were fixed in 4% PFA and prepared for histological analysis.

### Transient and stable transfections

Human podocytes (HPC) were grown as previously described [34]. HEK293 and NIH3T3 cells were cultured at 37°C/5% CO2 in complete DMEM medium. Cells were transiently transfected with Lipofectamine 2000 (Invitrogen) according to the manufacturer’s recommendations. For generation of stable GFP-Schip1-expressing HEK293-cell clones, cells were selected and maintained in medium containing 500 μg/ml of G418. Stable transfectants were characterized by Western blotting for GFP-Schip1 expression and other cell lines used in experiments were characterized for basal expression of the studied proteins (S3 Fig.).

### Immunofluorescence

Cryosections (5 μm thick) of human and mouse kidneys were postfixed with cold acetone at −20°C or with 4% paraformaldehyde (PFA) at room temperature. Cells were grown on fibronectin-coated (10 μg/ml) glass coverslips, fixed with 4% PFA for 20 min at room temperature, and subsequently permeabilized by incubation with 0.1% triton X-100/PBS for 5 min, followed by blocking with 2% BSA/PBS for 1 h. In some experiments, cells were treated with 0.5% saponin/PBS for 20 min before fixation. Immunofluorescence was performed as described previously [35]. For double labeling, incubations were performed sequentially to prevent cross-reactions. Photos were taken using Zeiss LSM510 microscope, with 20x, 40x or 63x objectives.

### Drug treatments

Cells were plated on fibronectin-coated coverslips in 24-well plates, and left to adhere and spread for 16 h. Thereafter, cells were incubated for 1 h at 37°C with 10 μg/ml of the actin monomer-sequestering drug latrunculin A, actin-polymerisation inhibiting drug cytochalasin D or microtubule disassembly drug nocodazole (all from Sigma). Control cells were incubated with the vehicle only. Alternatively, HEK293 and NIH3T3 cells (shown to express the PDGFβ receptors [36]) were treated with media containing 50 μg/ml PDGF-BB for 2 h at 37°C.

### Yeast two-hybrid screening

Yeast two-hybrid screen was performed as previously described using a mouse kidney glomerulus cDNA library [35,37]. The library was screened with baits encoding the Gal4 DNA-binding domain fused to the full-length Schip1 or its deletion variant, composed only of the most C-terminal coiled-coil domain. The screen with Schip1 coiled-coil domain bait resulted in 400 colonies initially picked for screening. Through several rounds of elimination of false positives on plates with selective drop-out medium, this number was reduced to 32 clones that were finally sequenced. Of these, 5 clones contained the full-length transcript of Nherf2.

### Western blotting and coimmunoprecipitations

Western blotting was carried out following standard procedures. For coimmunoprecipitations, confluent transiently transfected HEK293 cells or pig glomeruli were washed twice in cold PBS and lysed in 0.5% Triton X-100, 20 mM Tris-HCl (pH 7.4) and 150 mM NaCl buffer containing protease inhibitor cocktail (Roche Diagnostics, Mannheim, Germany) and phosphatase inhibitors (1mM NaVO_3_, 50 mM NaF). Lysates were clarified by centrifugation (14,000 ***g***), incubated with primary antibodies overnight, followed by an incubation with protein A+G agarose beads (Roche) for 1 h at 4°C. The beads were washed 3 times with the lysis buffer, resuspended in 1x SDS-sample loading buffer, and boiled for 10 min prior to SDS-PAGE analysis.

### Förster (Fluorescent) Resonance Energy Transfer (FRET) in fixed cells

A detailed description of the FRET technique can be found elsewhere [38]. The Förster constant (*R*0) for the donor-acceptor pair used in this study, Alexa488 and Alexa568, is 62Å. To determine FRET, we quantified the quenching of donor fluorescence by performing acceptor photobleaching. FRET measurements were performed using a Zeiss LSM510 inverted confocal microscope, Apochromat ×63/1.4 NA oil immersion objective and the Zeiss LSM510 software version 2.8. Briefly, fluorophores were excited with 488 and 543 nm lasers and images collected separately. The acceptor, Alexa568, was then irreversibly photobleached in a selected adequate region by continuous excitation with a 543 nm laser for about 30 s. Residual Alexa568 and Alexa488 image was obtained under the same settings as prebleach images, identical regions on individual cells were outlined in the photobleached area and processed using ImageJ. In a typical experiment, 15–20 cells were measured for each sample. As a positive control for FRET, we examined GFP-Schip1-expressing cells immunostained for the GFP using Alexa568-labeled IgG as a secondary antibody.

### Immunoelectron microscopy (IEM)

Samples from human renal cortexes were fixed in 3% PFA in 0.1M phosphate buffer. IEM experiments and semiquantification of the Schip1 signal were conducted in a blinded fashion, as described previously [35]. Single and double IEM was performed with Schip1 and ezrin/Nherf2 antibodies, labeled with 10 and 5 nm gold particles, respectively.

### Cell migration assay

Stably transfected GFP-Schip1 HEK293 cells were plated on fibronectin coated dishes in triplicates. Cells were grown to confluence and serum starved in 0.5% FCS overnight. Thereafter, monolayers were wounded by manual scratching with a 200-μl pipette tip, washed with PBS and incubated at 37°C in complete media with or without 50 μg/ml of PDGF-BB. At the indicated times, phase contrast images at 3 wound sites were captured by Leica brightfield microscope and analyzed by ImageJ. Results are reported in percent showing the size of the wound compared to the 0-point (right after scratching), representative of three experiments.

### *In vitro* actin polymerization and depolymerization assays

Assays were performed using the Actin Polymerization Kit (Biochem, Denver, CO; BK003). The assay relies on the difference between fluorescent signals of pyrene labeled monomeric G-actin and polymerized pyrene/F-actin (used as controls in our experiments). For these assays, we used lysates prepared from 293 cells stably expressing GFP–Schip1, and control cells. Cells were plated onto plates coated with 10 mg/ml of fibronectin, and allowed to adhere overnight in a serum-free media, followed by an overnight treatment with medium containing 10% fetal calf serum or 30 mg/ml of PDGFBB (Invitrogen, PHG0044). After this, cells were collected and lysed for 3 h at 41 C in a buffer containing 20 mmol/l Tris-HCl, pH 7.5, 20 mmol/l NaCl, and protease inhibitors. Lysates were clarified by centrifugation at 14,000 rpm for 10 min. Assays were performed according to the manufacturers instructions, and fluorescence kinetic measurements recorded with TECAN GENiosPro.

### Statistical analyses

In all experiments groups were compared by a non-parametric ANOVA or Student’s T-test. Public microarray datasets deposited in the Gene Expression Omnibus were used in the study (GSE21785, GSE39441, GSE33714). Statistical analyses were done with the GraphPad Prism 6 software and p-value <0.05 was considered to indicate significance.

## Supporting Information

S1 Fig Conservation of Schip1 primary protein structure among species. Analysis of human, mouse and zebrafish Schip1 amino-acid sequences by PRALINE multiple alignment program shows high level of protein identity among the species. The C-terminal half of Schip1, containing the functional domains, is nearly identical.
(TIF)

S2 Fig Additional evidence for Schip1 cell localization and actin association upon latrunculin A, cytochalasin D and nocodazole treatments. **(A)** Schip1 localizes to peripheral lamellipodia regions near the plasma membrane. This is shown both for the endogenously (arrowheads, upper panel) and ectopically (arrowheads, lower panel) expressed protein in human podocytes. **(B)** MycSchip1- transfected podocytes treated with latrunculin A or cytochalasin D before fixation and stained with anti-Myc antibodies and rhodamine-phalloidin show Schip1 association with disturbed F-actin fibers (upper panel, arrowheads) and preservation of cortical actin (lower panel, arrowheads). **(C)** In MycSchip1-transfected podocytes treated with nocodazole and stained with anti-Myc and tubulin antibodies, Schip1 localisation at the lamellipodia is not affected.
(TIF)

S3 Fig Additional evidence for Schip1-overexpressing cells response to PDGF-BB treatment, control RT-PCR and Western blots and extra photomicrographs. **(A)** Two extra fields of MycSchip1 transfected NIH3T3 cells, stimulated with PDGF-BB. Schip1 overexpression induces actin reorganization in response to PDGF-BB, cortical actin accumulation and dissolution of transversal parallel actin fibers (arrowheads). **(B)** Control RT-PCR and WBs showing expression of: cellular markers in freshly isolated glomerular fractions (Nphs1 for YFP+ podocytes, Ehd3 for endothelial cells in YFP- glomerular fraction, first panel); confirmation of GFP-Schip1 expression in stably transfected HEK293 cells (second panel); confirmation of Schip1 expression in MycSchip1 transfected HEK293 cells characterized by anti-Myc-tag and anti-Schip1 antibodies (third panel); basal endogenous levels of Schip1, ezrin and Nherf2 in cultured human podocytes (HPC), NIH3T3 and HEK293 cells (fourth panel). Immunoblotting for calnexin used as loading control. **(C)** Additional immunoelectron micrographs showing localization of Schip1 in the human glomerular filter. Schip1 is mostly detected in the podocytes, occasionally also in endothelial (EC) and mesangial cells (MC). **(D)** Double immunofluorescence stainings on human kidney sections of Schip1 with endothelial (CD31) and mesangial (alpha smooth muscle actin, aSMA) cell markers show limited signal overlap.
(TIF)

S4 Fig Whole blots for coimmunoprecipitations presented in Fig. 7. Blot presented in **(A)** corresponds to Fig. 7A upper panel, blot in **(B)** to Fig. 7A middle panel and blot in **(D)** to Fig. 7A lower panel. Blot in **(C)** represents additional evidence of the interaction between Schip1 and Ezrin in transfected cells. OE-overexpression by transfection, IP-immunoprecipitation, WB-Western blot.
(TIF)
