# Correction: Schip1 Is a Novel Podocyte Foot Process Protein that Mediates Actin Cytoskeleton Rearrangements and Forms a Complex with Nherf2 and Ezrin

**DOI:** 10.1371/journal.pone.0126079

**Published:** 2015-05-12

**Authors:** Ljubica Perisic, Patricia Q. Rodriguez, Kjell Hultenby, Ying Sun, Mark Lal, Christer Betsholtz, Mathias Uhlén, Annika Wernerson, Ulf Hedin, Timo Pikkarainen, Karl Tryggvason, Jaakko Patrakka

There are errors in the affiliations for authors Patricia Q. Rodriguez and Jaakko Patrakka. Both authors are affiliated with #1, Division of Matrix Biology, Department of Medical Biochemistry and Biophysics, Karolinska Institute, Stockholm, Sweden, and #2 KI/AZ Integrated CardioMetabolic Center, Department of Medicine, Karolinska Institutet at Karolinska University Hospital Huddinge, Stockholm, Sweden.
