# Supplementary figures and images for: Schip1 Is a Novel Podocyte Foot Process Protein that Mediates Actin Cytoskeleton Rearrangements and Forms a Complex with Nherf2 and Ezrin
Source: PLoS One. 2015 Mar 25;10(3):e0122067. doi: 10.1371/journal.pone.0122067 (PMC4373682; doi:10.1371/journal.pone.0122067)

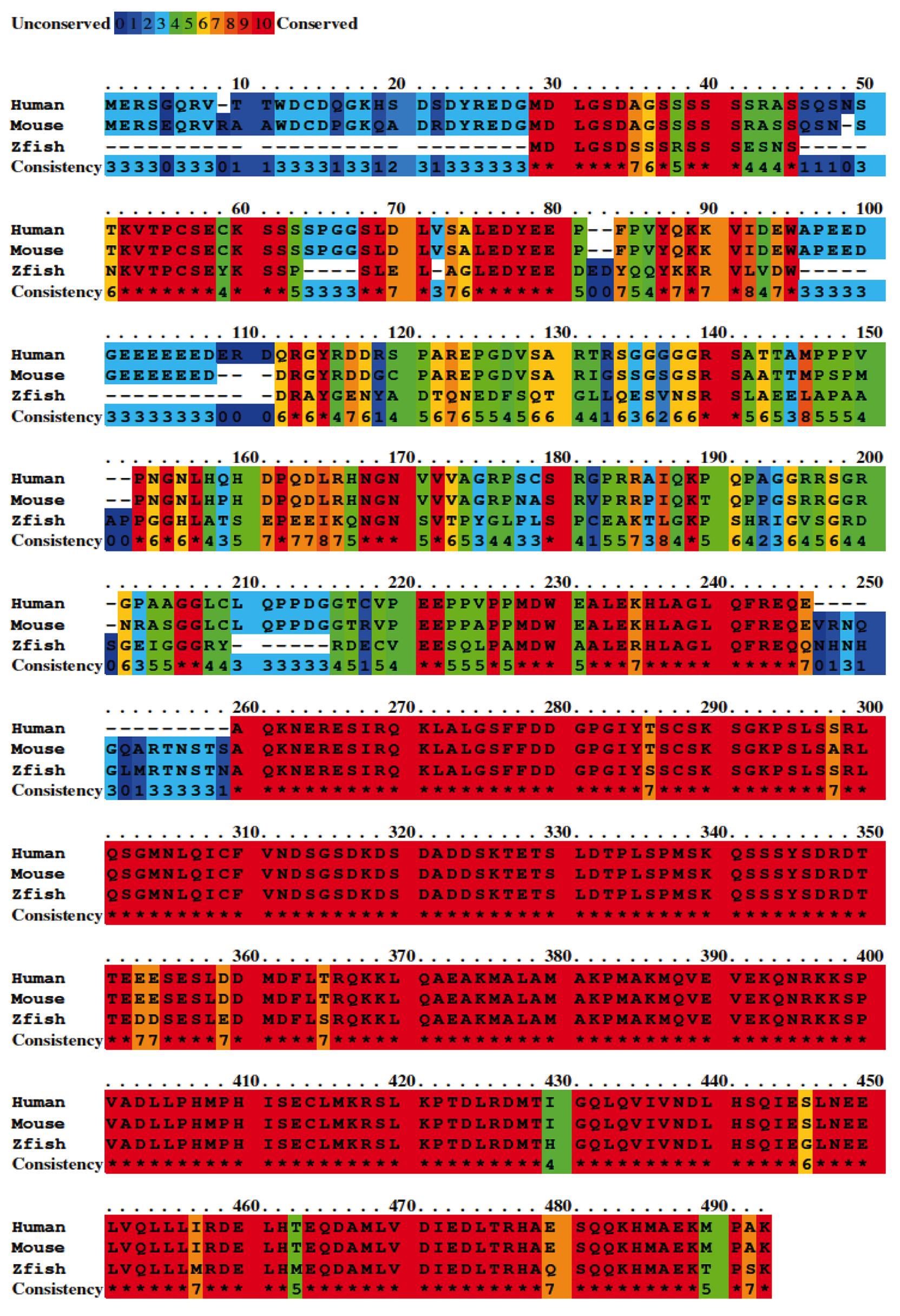

Supplement: S1 Fig — Analysis of human, mouse and zebrafish Schip1 amino-acid sequences by PRALINE multiple alignment program shows high level of protein identity among the species. The C-terminal half of Schip1, containing the functional domains, is nearly identical. (TIF) [file pone.0122067.s001.tif]

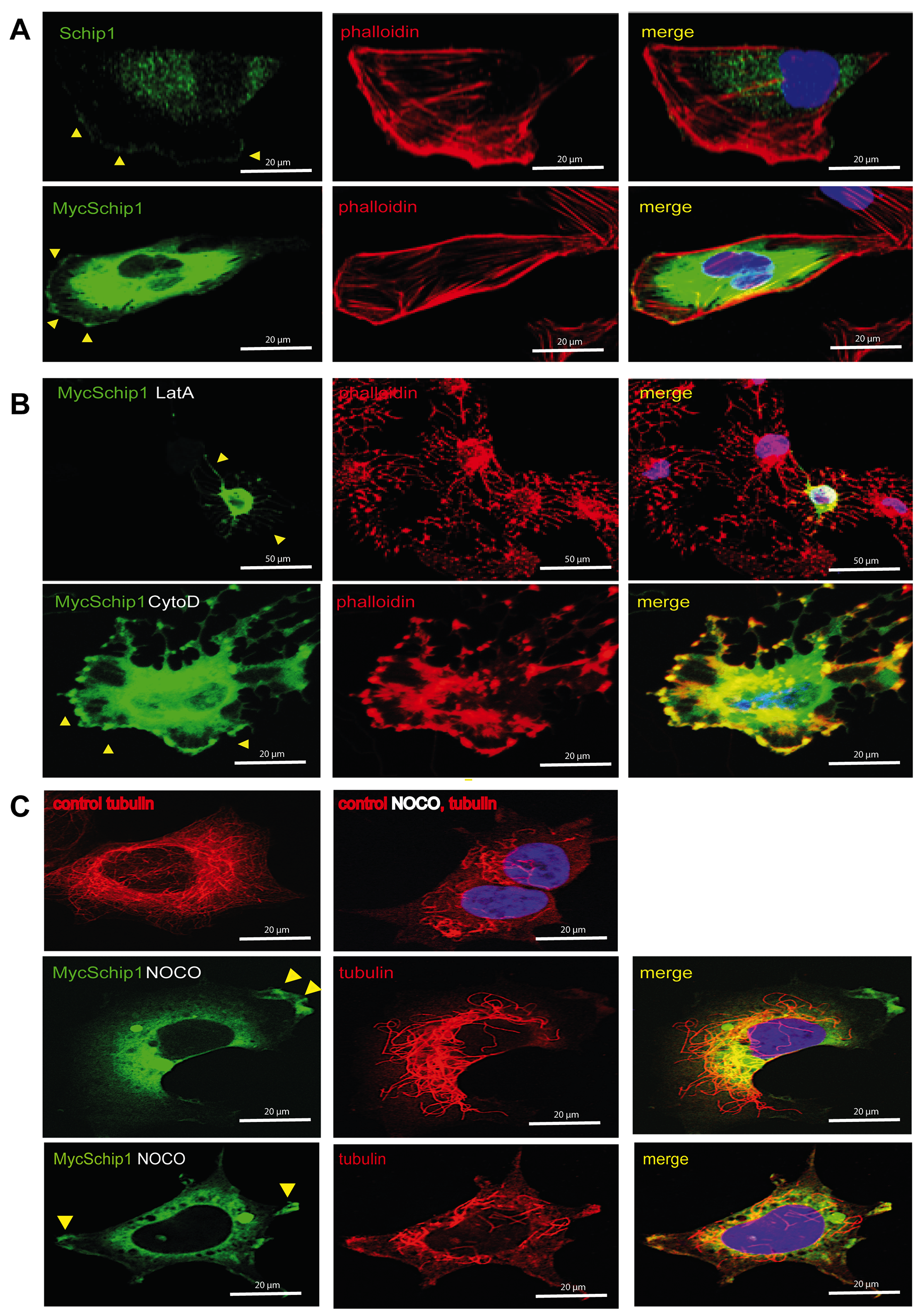

Supplement: S2 Fig — (A) Schip1 localizes to peripheral lamellipodia regions near the plasma membrane. This is shown both for the endogenously (arrowheads, upper panel) and ectopically (arrowheads, lower panel) expressed protein in human podocytes. (B) MycSchip1- transfected podocytes treated with latrunculin A or cytochalasin D before fixation and stained with anti-Myc antibodies and rhodamine-phalloidin show Schip1 association with disturbed F-actin fibers (upper panel, arrowheads) and preservation of cortical actin (lower panel, arrowheads). (C) In MycSchip1-transfected podocytes treated with nocodazole and stained with anti-Myc and tubulin antibodies, Schip1 localisation at the lamellipodia is not affected. (TIF) [file pone.0122067.s002.tif]

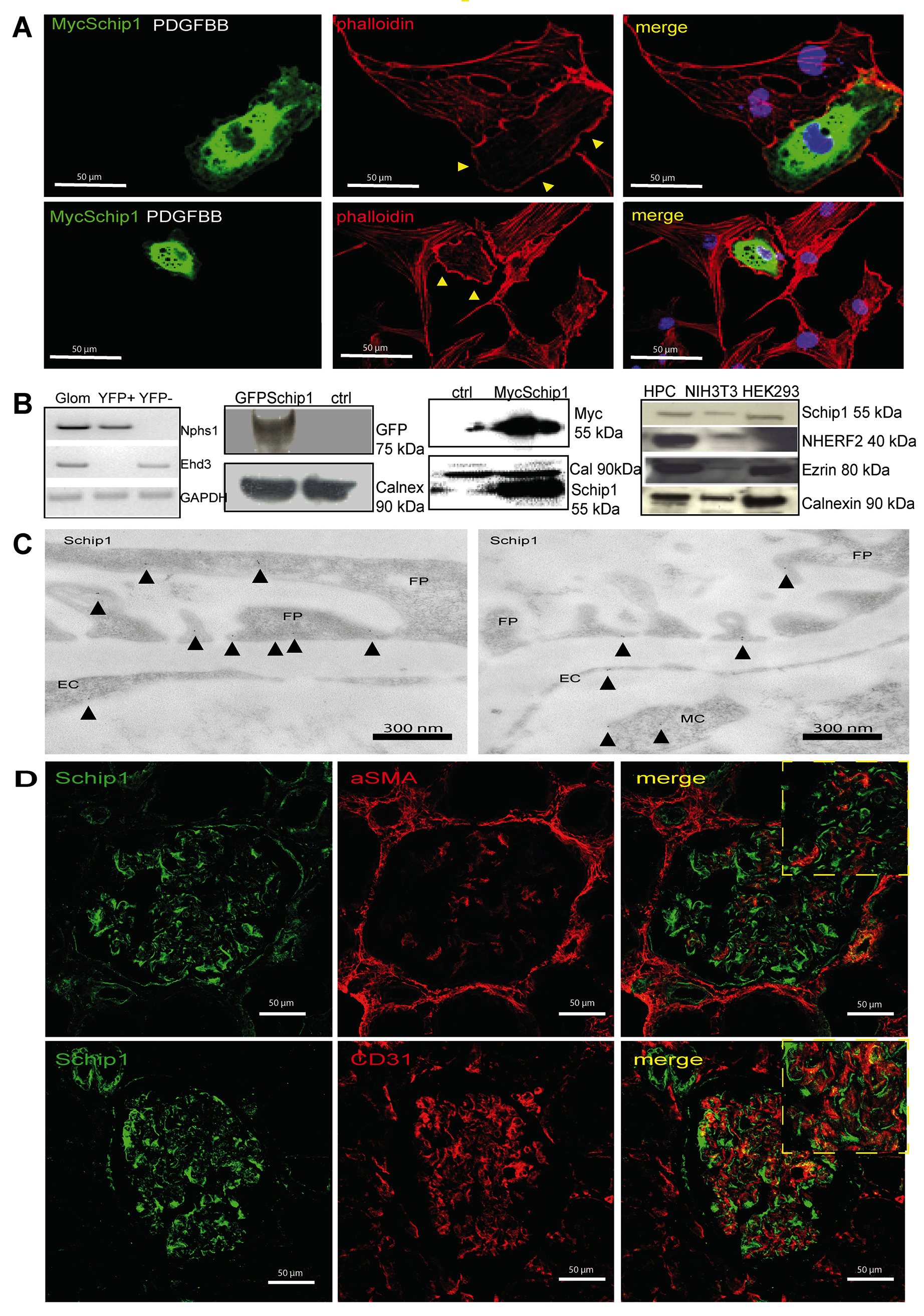

Supplement: S3 Fig — (A) Two extra fields of MycSchip1 transfected NIH3T3 cells, stimulated with PDGF-BB. Schip1 overexpression induces actin reorganization in response to PDGF-BB, cortical actin accumulation and dissolution of transversal parallel actin fibers (arrowheads). (B) Control RT-PCR and WBs showing expression of: cellular markers in freshly isolated glomerular fractions (Nphs1 for YFP+ podocytes, Ehd3 for endothelial cells in YFP- glomerular fraction, first panel); confirmation of GFP-Schip1 expression in stably transfected HEK293 cells (second panel); confirmation of Schip1 expression in MycSchip1 transfected HEK293 cells characterized by anti-Myc-tag and anti-Schip1 antibodies (third panel); basal endogenous levels of Schip1, ezrin and Nherf2 in cultured human podocytes (HPC), NIH3T3 and HEK293 cells (fourth panel). Immunoblotting for calnexin used as loading control. (C) Additional immunoelectron micrographs showing localization of Schip1 in the human glomerular filter. Schip1 is mostly detected in the podocytes, occasionally also in endothelial (EC) and mesangial cells (MC). (D) Double immunofluorescence stainings on human kidney sections of Schip1 with endothelial (CD31) and mesangial (alpha smooth muscle actin, aSMA) cell markers show limited signal overlap. (TIF) [file pone.0122067.s003.tif]

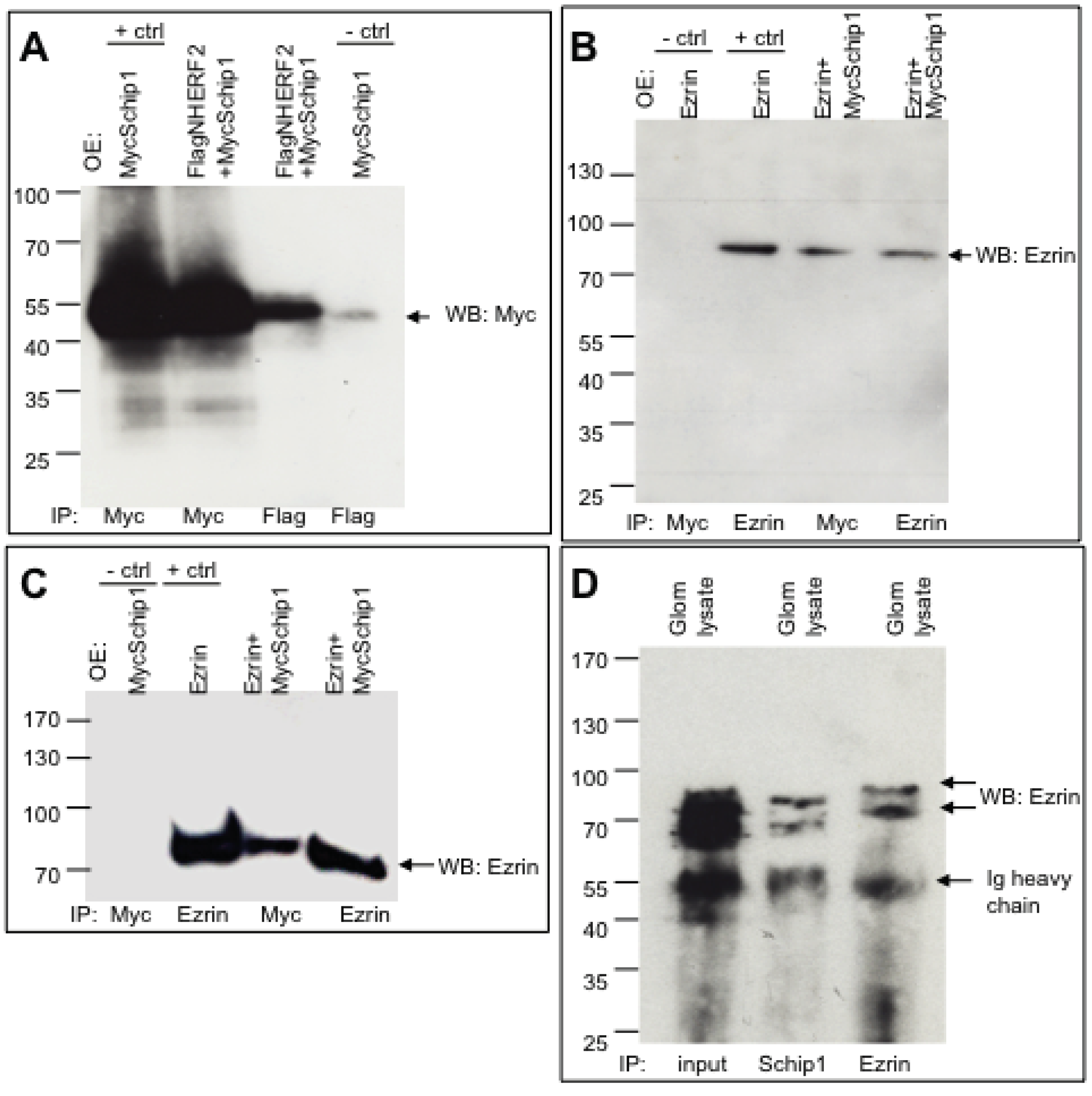

Supplement: S4 Fig — Blot presented in (A) corresponds to Fig. 7A upper panel, blot in (B) to Fig. 7A middle panel and blot in (D) to Fig. 7A lower panel. Blot in (C) represents additional evidence of the interaction between Schip1 and Ezrin in transfected cells. OE-overexpression by transfection, IP-immunoprecipitation, WB-Western blot. (TIF) [file pone.0122067.s004.tif]
